# Rising Threats and Evolving Trends: Five Years of Urinary Tract Infection Prevalence in a Portuguese Hospital

**DOI:** 10.3390/clinpract15060100

**Published:** 2025-05-26

**Authors:** Francisco José Barbas Rodrigues, Patrícia Coelho, Sónia Mateus, Miguel Castelo-Branco

**Affiliations:** 1 Sport Physical Activity and Health Research & Innovation Center (Sprint), Polytechnic Institute of Castelo Branco, 6000-084 Castelo Branco, Portugal; patriciacoelho@ipcb.pt (P.C.); soniamateus@ipcb.pt (S.M.); 2 Faculty of Health Sciences, The University of Beira Interior, 6201-001 Covilha, Portugal; mcbranco@fcsaude.ubi.pt

**Keywords:** urinary tract infections (UTIs), antibiotic resistance, antibiotic stewardship, epidemiology of UTIs, antimicrobial consumption, Portugal

## Abstract

**Background/Objective:** Urinary tract infections (UTIs) are a significant public health concern worldwide, yet longitudinal data from Portuguese hospital settings remain limited. This study aimed to characterize epidemiological trends, microbial etiology, antimicrobial resistance patterns, and associated risk factors of UTIs over a five-year period (2018–2022) in a central Portuguese hospital. **Methods:** In this retrospective observational study, 23,682 positive urine cultures were analyzed from specimens collected between January 2018 and December 2022. Data were extracted from the laboratory information system and included patient demographics, clinical service of origin, isolated microorganisms, resistance profiles, and annual antibiotic consumption (Defined Daily Dose (DDD) per 1000 patient-days). UTI prevalence was calculated as the proportion of positive cultures among all urine samples processed annually. **Results:** The positivity rate increased from 18.7% in 2018 to 22.7% in 2022, with a peak in 2019. Women represented around 70% of cases throughout the study period. Most infections originated from inpatient wards, followed by emergency services. *Escherichia coli* remained the leading pathogen (≈62%), followed by *Klebsiella pneumoniae* (≈14%) and *Enterococcus faecalis* (≈8%). Risk factors included catheterization (37.2%), prior UTI history (22.1%), and diabetes mellitus (18.5%). Longer hospital stays (>7 days) were associated with increased positivity. For *E. coli*, resistance ranged from 2% (amikacin) to 41% (ampicillin), with increasing resistance to ertapenem and fosfomycin and decreasing resistance to several key antibiotics. *K. pneumoniae* showed 4–36% resistance across antimicrobials, with notable increases for fosfomycin, meropenem, and cefuroxime axetil. Antibiotic usage trends reflected these patterns, with declining use of amikacin and rising use of cefuroxime axetil and meropenem. **Conclusions:** Over the five-year period, both UTI prevalence and resistance to critical antimicrobials increased, reinforcing the need to update empirical treatment guidelines. Identified risk factors may inform targeted prevention strategies. Ongoing surveillance and antimicrobial stewardship are crucial to mitigate the rising burden of UTIs and resistance

## 1. Introduction

Urinary tract infections (UTIs) are among the most prevalent infectious diseases worldwide, representing a significant public health concern, particularly in terms of incidence [1]. These infections occur when pathogenic microorganisms colonize different parts of the urinary system, such as the kidneys, bladder, ureters, and urethra. Although the normal urethral microbiota plays a protective role against microbial invasion, alterations in this ecosystem may favor the proliferation of infectious agents, leading to the development of UTI [2,3]. Female anatomy, characterized by a shorter urethra and its proximity to the perianal region, contributes to the increased vulnerability of women, as it facilitates the ascent of uropathogenic pathogens [4]. The most frequent uropathogens include *Escherichia coli*, *Klebsiella pneumoniae*, *Enterococcus faecalis*, and *Proteus mirabilis*. First-line antimicrobials for uncomplicated UTIs often comprise nitrofurantoin, fosfomycin, and trimethoprim–sulfamethoxazole, while fluoroquinolones and third-generation cephalosporins are reserved for complicated cases [4,5].

Studies indicate that up to 5% of urine samples with positive bacterial growth may be associated with asymptomatic colonization, which can evolve into an active infection under certain conditions [5]. The risk of developing UTIs is influenced by various factors, such as genetic predisposition, chronic comorbidities, hormonal variations, a history of recurrent infections, structural anomalies of the urinary tract, and the use of invasive devices, such as catheters [6]. Early detection is crucial to prevent more severe complications, such as pyelonephritis and permanent renal damage [7]. Clinical evaluation is primarily based on typical signs, such as dysuria, increased urinary frequency, and suprapubic pain, while diagnostic confirmation involves laboratory urine analysis and microbiological culture, allowing the identification of the pathogen and its resistance profile [8].

The choice of appropriate treatment should consider the microbiology of the infection and the sensitivity to antibiotics, being essential to ensure therapeutic efficacy and minimize antimicrobial resistance. In more urgent cases, empirical treatment is commonly adopted, based on local patterns of bacterial resistance [9]. The advancement of laboratory techniques, such as DNA amplification by PCR, has facilitated faster and more accurate diagnoses, optimizing clinical management and contributing to a more rational use of antimicrobials [10].

Despite the clinical importance of urinary tract infections (UTIs), longitudinal data on their epidemiology and resistance trends remain limited in Portugal. Most published studies focus on short-term analyses or are restricted to specific populations or geographic regions. Moreover, the COVID-19 pandemic may have significantly impacted antimicrobial consumption and resistance dynamics, reinforcing the need for updated, comprehensive data from hospital settings. In Portugal, few studies have evaluated UTI trends over extended periods, limiting the capacity to inform empirical treatment protocols and resistance containment strategies. By providing long-term data, this study supports evidence-based policy and stewardship interventions.

This study aims to expand existing knowledge by characterizing the sociodemographic profile of patients with positive urine cultures between 2018 and 2022 in a Hospital Center in Portugal, identifying the main bacterial agents isolated and evaluating the evolution of their susceptibility to antimicrobials over the analyzed period.

## 2. Materials and Methods

This retrospective observational study analyzed all positive urine cultures collected between January 2018 and December 2022 at a hospital center located in central Portugal, comprising a total of 23,682 samples.

### 2.1. Study Population and Inclusion Criteria

All urine samples from patients of all ages and both sexes processed by the microbiology laboratory during the study period were considered. Only cultures with a monomicrobial or clinically significant polymicrobial results were included. Duplicate samples (i.e., multiple samples from the same patient within a 7-day interval) were excluded to avoid overrepresentation of individual cases. A culture was considered positive when it presented a bacterial count ≥ 10^5^ colony-forming units per milliliter (CFU/mL), in line with accepted clinical microbiology standards.

### 2.2. Data Collection and Variables

Data were extracted from the laboratory information system (LIS) using anonymized identifiers and included:

Demographics: sex (male/female) and age (in years);

Admission type: emergency department (ED), inpatient ward (>24 h), outpatient visit, or day hospital;

Microbiological data: isolated microorganism(s) and antimicrobial susceptibility profile (resistant/sensitive);

Clinical context: presence of urinary catheter, history of previous UTI (within the past 12 months), and diagnosis of diabetes mellitus.

The data were compiled into a master database for analysis.

### 2.3. Microbiological Methods

Urine specimens were cultured on chromogenic agar plates (bioMérieux, Marcy-l’Étoile, France) and incubated for 18–24 h at 37 °C. Identification of bacterial species was performed using the VITEK^®^ 2 system (bioMérieux), with confirmation by MALDI-TOF MS (matrix-assisted laser desorption/ionization time-of-flight mass spectrometry—Bruker Daltonics, Bremen, Germany) when necessary. Antimicrobial susceptibility testing was performed using broth microdilution via VITEK^®^ 2 AST cards. In cases of discordant or intermediate results, E-tests were employed. Susceptibility interpretation followed the EUCAST guidelines valid at the time of each analysis. Notably, fosfomycin susceptibility was interpreted exclusively for *E. coli*, as per EUCAST recommendations.

### 2.4. Antibiotic Consumption Data

Annual data on institutional antibiotic consumption were obtained from the Portuguese National Health Service (SNS) open-access drug utilization databases. Antibiotic usage was expressed in Defined Daily Doses (DDD) per 1000 patient-days, in accordance with the World Health Organization’s ATC/DDD classification.

### 2.5. Statistical Analysis

Descriptive statistics were performed using IBM SPSS Statistics for MacOS (version 29.0.1). Categorical variables were described using frequencies and percentages, and numerical variables using means and standard deviations. No imputation of missing data was performed. Figures were produced in Microsoft Excel and SPSS.

### 2.6. Ethical Considerations

This study was approved by the Ethics Committee and Data Protection Officer of the University of Beira Interior (approval no. CE-UBI-Pj-2023-020; issued on 19 April 2023). Given its retrospective nature and the use of anonymized data, the requirement for informed consent was waived.

This study is part of the ITUCIP project (Urinary Tract Infections in Central Portugal) and aims to contribute to the evidence base for clinical decision-making and infection control policies in hospital settings.

## 3. Results

**Figure 1:**
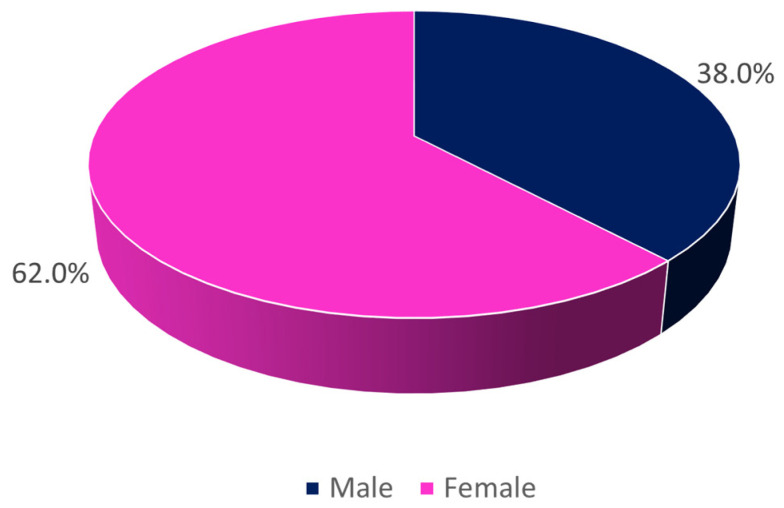
Distribution of the sample by sex (*n* = 23,682).

A total of 23,682 urine cultures were analyzed, corresponding to all positive urine samples for bacteria that entered into this institution between January 2018 and December 2022 (60 months), with the majority being female (Figure 1).

Of these, 19.3% correspond to the year 2018, 19.7% to 2019, 18.2% to 2020, 20.1% to 2021, and 22.7% to the year 2022. These percentages represent the share of positive cultures among all urine specimens processed each year, rising from 19.3% in 2018 to 22.7% in 2022, indicating a clear upward trend in culture positivity.

**Figure 2:**
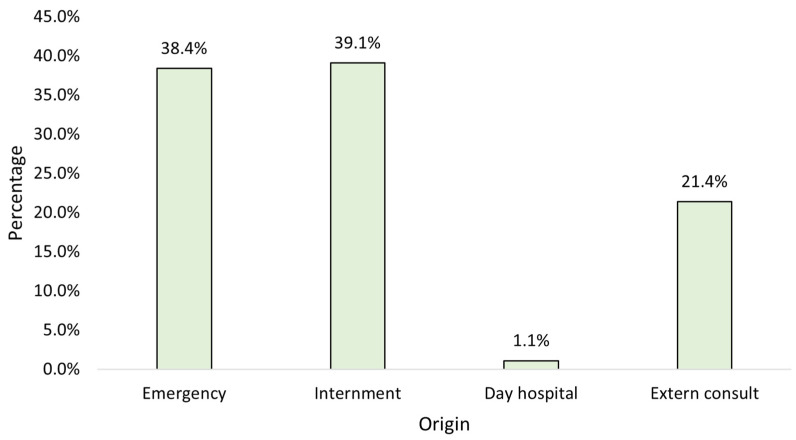
Distribution of the sample by origin (*n* = 23,682).

When studying the strains by origin, it is observed that the majority originated from inpatient admissions, followed closely by emergency admissions (Figure 2).

**Figure 3:**
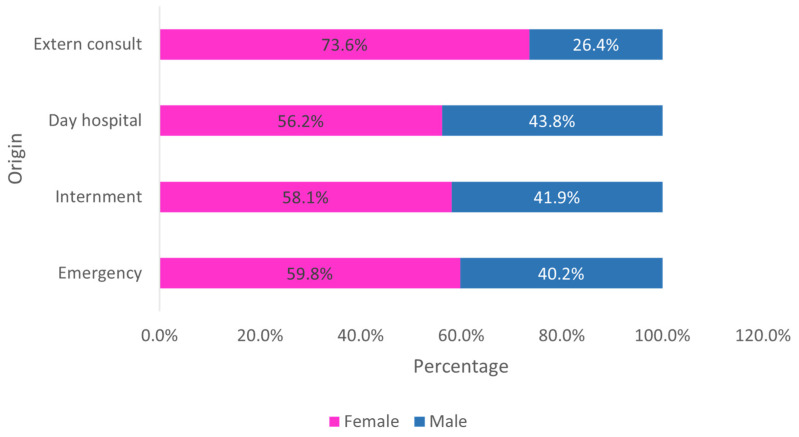
Distribution of the sample by sex and origin (*n* = 23,682).

When analyzing the origin by sex, it is observed that women are always in greater number, regardless of the origin (Figure 3).

**Figure 4:**
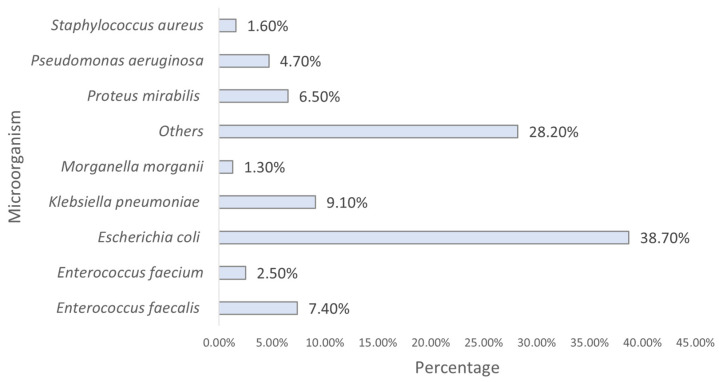
Distribution of the sample by strain (*n* = 23,682). Others include all other species with <2% prevalence each.

Analyzing the identified strains, we found that *E. coli* was the most predominant, followed by *K. pneumoniae* and *E. faecalis* (Figure 4).

**Figure 5:**
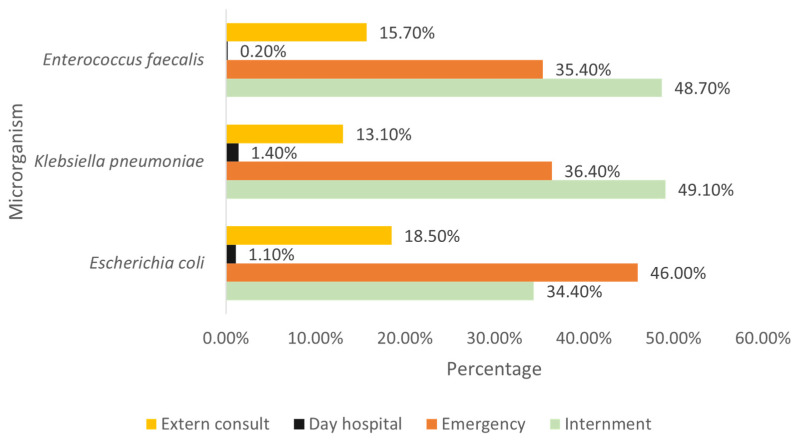
Distribution of the most prevalent strains by origin.

When observing the main strains identified by origin, it is found that the majority of *E. coli* originate from emergency patients, while most *K. pneumoniae* come from inpatient admissions, as well as the majority of *E. faecalis* (Figure 5).

**Figure 6:**
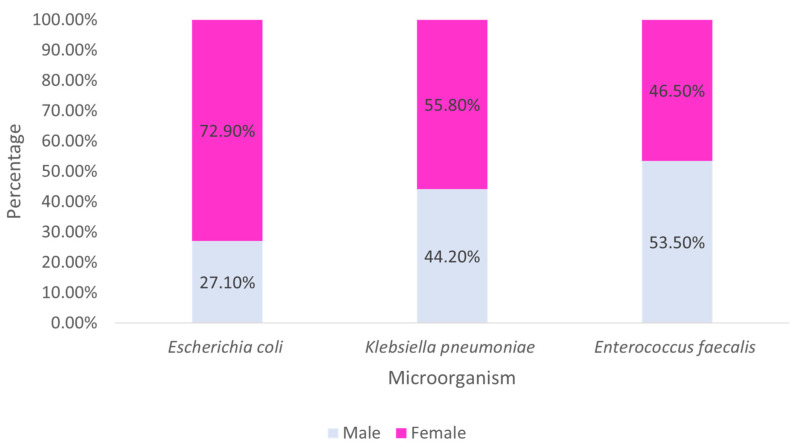
Distribution of the most prevalent strains by sex.

Examining the main strains found by sex, it is observed that the majority of *E. coli* and *K. pneumoniae* were identified in females, while the majority of *E. faecalis* came from males (Figure 6).

An analysis of risk factors revealed that patients with prolonged hospital stays (>7 days), urinary catheterization, or a documented history of UTI in the preceding year had a significantly higher prevalence of positive urine cultures. Specifically, catheterized patients accounted for 37.2% of all positive samples, with a higher proportion of infections caused by *K. pneumoniae* and *E. faecalis*. These findings support the hypothesis that nosocomial exposure and invasive devices are associated with increased UTI risk.

Relating the strain found (among the three most prevalent) with the origin and sex, it is observed that both *E. coli* and *K. pneumoniae* are always more frequently found in females, regardless of the origin. On the other hand, *E. faecalis* predominates in males, originating from inpatient admissions, day hospital, and emergency, with the majority of *E. faecalis* samples from outpatient consultations being from females.

The heterogeneity of resistance profiles varied considerably among the three main pathogens. For *E. coli*, resistance rates to antibiotics ranged from 2% (amikacin) to 41% (ampicillin), while for *K. pneumoniae* the resistance varied from 4% (amikacin) to 36% (cefuroxime axetil). *E. faecalis* showed resistance rates from 3% (linezolid) to 28% (ciprofloxacin). This diversity underscores the complexity of empirical treatment decisions in clinical settings.

Examining the behavior of the two most prevalent bacteria in relation to the antibiotics tested at Unit B, specifically the percentage of resistant strains per year, we observed that *E. coli* increased its resistance to two antibiotics (Ertapenem and Fosfomycin) between 2018 and 2022, decreased resistance to six antibiotics (Amikacin, Ampicillin, Amoxicillin/Clavulanic acid, Cefepime, Gentamicin, and Piperacillin/Tazobactam), and remained stable against one antibiotic (Cefuroxime). *K. pneumoniae* saw an increase in resistance to three antibiotics (Fosfomycin, Meropenem, and Cefuroxime Axetil) between 2018/2022, decreased resistance to five antibiotics (Amikacin, Amoxicillin/Clavulanic acid, Cefepime, Ertapenem, Gentamicin), and remained stable against two antibiotics (Ampicillin, Piperacillin/Tazobactam).

**Figure 7:**
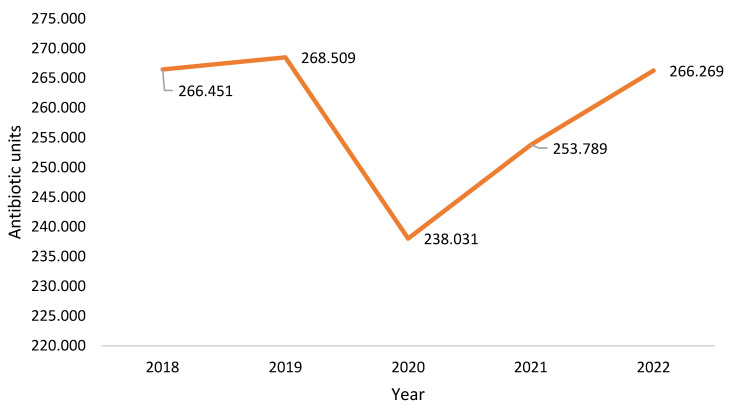
Antibiotic units consumed in the institution.

Antibiotic consumption in this healthcare unit between 2018 and 2022 is shown in Figure 7. In 2022, antibiotic consumption had not yet reached the levels of 2019 (pre-pandemic).

## 4. Discussion

The results of this study clearly demonstrate the widespread occurrence of urinary tract infections (UTIs) among humans, justifying their high prevalence [11]. The microbiological analysis revealed the predominant presence of *E. coli*, followed by other Gram-negative bacteria, such as *K. pneumoniae* and *P. mirabilis*, in accordance with the literature that identifies *E. coli* as the main etiological agent of both community-acquired and hospital-acquired UTIs [12]. The predominance of *E. coli* can be largely attributed to its ability to adhere to the urinary tract mucosa via type 1 and P fimbriae, thereby facilitating colonization and subsequent infection. Moreover, the metabolic versatility of this bacterium and its aptitude for acquiring resistance genes further reinforce its relevance in the context of UTIs [13].

Antimicrobial resistance has emerged as a critical factor, particularly with respect to third-generation cephalosporins and fluoroquinolones, and its evolution over the past five years in our setting presents specific patterns that warrant closer attention, especially in inpatient and ICU contexts [14]. Notably, we observed a sustained increase in resistance of *K. pneumoniae* to cefuroxime axetil and meropenem, two antibiotics often used in severe hospital-acquired infections. This may suggest selective pressure in more intensive care environments, which should be carefully monitored. The observed decline in resistance to agents such as amikacin and cefepime among *E. coli* may also reflect shifts in prescribing practices or improved stewardship policies implemented post-2020 [15,16,17].

Additionally, our analysis revealed a strong correlation between key risk factors and the incidence of UTIs. Patients with catheter use, prolonged hospital stays, or recurrent infection history had significantly higher rates of infection, particularly with multidrug-resistant organisms. These findings align with existing literature and reinforce the need for strict infection control in hospitalized populations [18]. Immunocompromised patients, such as those with diabetes mellitus or those undergoing immunosuppressive therapies, also showed increased vulnerability. Furthermore, biofilm formation on the surfaces of urinary catheters represents an additional challenge, as it provides protection to bacteria against antimicrobial agents and the host’s immune response. Therefore, strategies to reduce the duration of catheter use and effective infection control measures are imperative to decrease the incidence of hospital-acquired UTIs [18,19]. The evolution of resistance profiles over the five-year period shows dynamic patterns. For example, resistance to meropenem among *K. pneumoniae* rose from 12% in 2018 to 21% in 2022, while amikacin resistance dropped from 9% to 3% in *E. coli* over the same period. These trends likely reflect the changing antibiotic policies and clinical practices in the post-pandemic context.

In addition to the points already discussed, it is observed that the high prevalence of *E. coli* can also be explained by its adaptability to various ecological niches, which facilitates its persistence both in the environment and within the host [20]. The heterogeneity of resistance profiles among the different bacteria encountered underscores the need for individualized therapeutic approaches, considering each patient’s clinical history [21,22,23]. The variability in resistance patterns further suggests that local factors, such as the indiscriminate use of antibiotics and the inadequate implementation of infection control protocols, may be contributing to the current scenario [24]. The impact of the COVID-19 pandemic, which altered prescribing habits and antimicrobial consumption, also deserves consideration, as it may have influenced the resistance rates observed in recent years [25]. The association between prolonged use of invasive devices, such as catheters, and the increased incidence of UTIs reinforces the importance of prevention policies that include the continuous review of the procedures for insertion and maintenance of these devices [19]. Moreover, the role of biofilm in protecting microorganisms exposes a significant vulnerability in the efficacy of conventional treatments, necessitating the development of new antimicrobial strategies [26]. Comparative studies among different hospital centers indicate that the implementation of antibiotic stewardship programs can significantly reduce the prevalence of resistant strains [27,28]. The integration of epidemiological data with genetic analyses of pathogens may provide deeper insights into the mechanisms of resistance and transmission, guiding more effective interventions [25]. Collaboration among multidisciplinary teams, involving physicians, microbiologists, and pharmacists, is crucial for formulating therapeutic guidelines that minimize the risks associated with antimicrobial resistance [29]. Finally, the continuity of epidemiological monitoring, coupled with translational research, is essential to anticipate trends and adapt clinical practices, thereby improving outcomes for patients affected by UTIs. Moreover, the role of ICUs as hotspots for antimicrobial resistance emergence and dissemination is particularly relevant. Although our dataset did not differentiate ICU admissions from general inpatient care, the predominance of multidrug-resistant *K. pneumoniae* among hospitalized patients strongly suggests a need for targeted surveillance in intensive care units. Future studies should include more granular data from ICU settings to better understand resistance dynamics in critically ill populations.

## 5. Conclusions

This study demonstrated the high prevalence of urinary tract infections, underscoring the importance of integrated approaches for their control and management. The results confirm that *E. coli* is the primary etiological agent, attributed to its ability to adhere to the urinary tract mucosa and its metabolic versatility, which facilitate colonization and subsequent infection. The growing antimicrobial resistance, particularly regarding third-generation cephalosporins, fluoroquinolones, and other beta-lactams, reinforces the urgent need to revise empirical prescribing protocols and to implement robust antimicrobial control strategies.

The significant association between risk factors—such as prolonged hospital stay, the use of catheters, and a history of previous infections—and the incidence of UTIs highlights the importance of preventive measures, including reducing the duration of invasive device use and rigorously controlling biofilm formation. Additionally, the heterogeneity of resistance profiles among pathogens underscores the need for individualized therapeutic approaches, based on epidemiological data and genetic analyses that allow a deeper understanding of the mechanisms of resistance and transmission.

The continuity of epidemiological monitoring, coupled with the implementation of antibiotic stewardship programs and collaboration among multidisciplinary teams, is essential to adapt clinical practices and improve outcomes for patients affected by UTIs. Compared to recent European reports, our *E. coli* resistance rates to third-generation cephalosporins (ranging 12–18%) are slightly lower than those described in a Spanish multicenter study (15–22%), whereas meropenem resistance in *K. pneumoniae* (12–21%) parallels data from Italian reference centers (10–24%). These comparisons highlight regional nuances and emphasize the need for tailored stewardship programs in mid-sized hospitals in central Portugal. These efforts are crucial for mitigating the evolution of antimicrobial resistance and reducing the impact of these infections on public health.

## Data Availability

Due to ethical restrictions and the protection of participants’ confidentiality, the data are not publicly available. However, the datasets used and/or analyzed during the current study are available from the corresponding author on reasonable request.
